# 

**Published:** 1876-04

**Authors:** 


### Editorial.

The International Medical Congress will be formally opened in Philadelphia, at noon, on Monday, the 4th day of September, 1876, in the hall of the University of Pennsylvania. The public dinner will be given on Thursday, 7th September, at 6:30 P.M. The registration fee will be ten dollars, which entitles the member to a copy of the printed transactions of the Congress. The following addresses will be delivered in general meeting: Medicine, by Austin Flint, New York: Hygiene, by Henry I. Bowdich, Boston; Surgery, by Paul F. Eve, Nashville; Obstetrics, by Theophilus Parvin, Indianapolis; Chemistry, by Theodore G. Wormley, Columbus, Ohio; Medical Biography, by J. M. Toner, Washington; Address, by Dr. Harman Lebert, Breslau; Medical Education, by Nathan S. Davis, Chicago; Medical Literature, by Lunsford P. Yandell, Louisville; Mental Hygiene, by Jno. P. Gray, Utica, N.Y.; Medical Jurisprudence, by Stanford E. Chaille, New Orleans.

The discussions in the dictions will be opened as follows: In *Medicine*, by J. J. Woodward, U. S. A.; J. Lewis Smith, New York; Roberts Bartholow, Cincinnati; Charles Denison, Denver, Colorado. In *Biology*, by Christopher Johnson, Baltimore; J. W. S. Arnold, New York; Austin Flint, Jr., New York; Harrison Allen, Philadelphia. In *Surgery*, by John T. Hodgen, St. Louis; William H. Van Buren, New York; Lewis A. Sayre, New York; Claudius H. Mastin, Mobile. In *Dermatology and Syphilography*, by James C. White, Boston; Lucius Duncan Bulkley, New York; Freeman J. Bumstead, New York; E. L. Keyes, New York. In *Obstetrics*, by William H. Byford, Chicago; William Goodell, Philadelphia; Washington L. Atlee, Philadelphia; William T. Lusk, New York. In *Ophthalmology*, by Henry W. Williams, Boston; Herman Knapp, New York; E. Williams, Cincinnati; E. G. Loring, New York. In *Otology*, by Albert H. Buck, New York; Clarence J. Blake, Boston; H. N. Spencer, St. Louis. In *Sanitary Science*, by John H. Ranch, Chicago; Stephen Smith, New York; J. M. Woodworth, U. S. Marine Hospital Service; Thomas E. Satterthwaite, New York. In *Mental Diseases*, by Walter H. Kempster, Oshkosh, Wis.; Isaac Ray, Philadelphia; C. H. Hughes, St. Louis; C. H. Nichols, Washington.

The Medical Association of Georgia will convene in its 27th Annual Session, in Augusta, April 19th. The railroads will pass members at half rates.

DeSaussure Ford, President.

J. T. Johnson, Secretary.

As will be seen from the above official notice the Medical Association of Georgia convenes on Wednesday, the 19th of April. A large attendance is expected, and the profession of Augusta is prepared to do the handsome thing. Above all things let each member go to Augusta with the determination to make some permanent arrangement by which the valuable papers that are presented, from year to year, to the Association shall be published in a handsome volume. If this is not done, or some plan adopted by which these papers will be given to the profession, we predict that the interest in the Association will be, to a great extent, lost, and will dwindle into a mere social gathering, as but few will be disposed to give the labor necessary to get up valuable papers when there is no means adopted to give them to the profession at large.

---

Dr. Ralph Walsh's Combined Call Book and Tablet.—Of all the call books and physician's theory that we have seen this one more perfectly fills the "*indication*" than all others. In the first place, its size is more convenient; it is longer and much thinner than those in use. Then the dates are so arranged that it may be used until full, commencing at any time during the year. The tablet is certainly quite an addition. In addition to all the information in regard to antidotes, the rare or poisonous remedies, he gives a complete list with doses of all the important remedies. This valuable little book can be had by application to Dr. Ralph Walsh, Washington, D. C. Price, $1 50.

